# Multifactorial resistance mechanisms associated with resistance to ceftazidime-avibactam in clinical *Pseudomonas aeruginosa* isolates from Switzerland

**DOI:** 10.3389/fcimb.2023.1098944

**Published:** 2023-04-25

**Authors:** Baharak Babouee Flury, Anja Bösch, Valentin Gisler, Adrian Egli, Salome N. Seiffert, Oliver Nolte, Jacqueline Findlay

**Affiliations:** ^1^ Medical Research Center, Kantonspital St. Gallen, St. Gallen, Switzerland; ^2^ Division of Infectious Diseases and Hospital Epidemiology, Kantonsspital St. Gallen, St. Gallen, Switzerland; ^3^ Department of Infectious Diseases, Inselspital, Bern University Hospital, University of Bern, Bern, Switzerland; ^4^ Clinic of Infectious Diseases and Hospital Hygiene, Kantonsspital Aarau, Aarau, Switzerland; ^5^ Department of Microbiology, Institute for Laboratory Medicine, Kantonsspital Aarau, Aarau, Switzerland; ^6^ Institute of Medical Microbiology, University of Zurich, Zurich, Switzerland; ^7^ Division of Human Microbiology, Centre for Laboratory Medicine, St. Gallen, Switzerland; ^8^ Medical and Molecular Microbiology, Faculty of Science and Medicine, University of Fribourg, Fribourg, Switzerland

**Keywords:** *Pseudomonas aeruginosa*, ceftazidime-avibactam (CZA), molecular resistance mechanisms, imipenem, whole genome sequencing (WGS)

## Abstract

**Background:**

Increasing reports of multidrug resistance (MDR) in clinical *Pseudomonas aeruginosa* have led to a necessity for new antimicrobials. Ceftazidime-avibactam (CZA) is indicated for use against MDR *P. aeruginosa* across a broad range of infection types and particularly those that are carbapenem resistant. This study sought to determine the molecular mechanisms of CZA and imipenem (IPM)-resistance in clinical *P. aeruginosa* isolates obtained from Swiss hospitals.

**Methods:**

Clinical *P. aeruginosa* isolates were obtained from inpatients in three hospitals in Switzerland. Susceptibility was determined by either antibiotic disc testing or broth microdilution according to EUCAST methodology. AmpC activity was determined using cloxacillin and efflux activity was determined using phenylalanine-arginine β-naphthylamide, in agar plates. Whole Genome Sequencing was performed on 18 clinical isolates. Sequence types (STs) and resistance genes were ascertained using the Centre for Genomic Epidemiology platform. Genes of interest were extracted from sequenced isolates and compared to reference strain *P. aeruginosa* PAO1.

**Results:**

Sixteen different STs were identified amongst the 18 isolates in this study indicating a high degree of genomic diversity. No carbapenemases were detected but one isolate did harbor the ESBL *bla*_PER-1_. Eight isolates were CZA-resistant with MICs ranging from 16-64 mg/L, and the remaining ten isolates had either low/wildtype MICs (n=6; 1-2 mg/L) or elevated, but still susceptible, MICs (n=4; 4-8 mg/L). Ten isolates were IPM-resistant, seven of which had mutations resulting in truncations of OprD, and the remaining nine IPM-susceptible isolates had intact *oprD* genes. Within CZA-R isolates, and those with reduced susceptibility, mutations resulting in *ampC* derepression, OprD loss, *mexAB* overexpression and ESBL (*bla*_PER-1_) carriage were observed in various combinations and one harbored a truncation of the PBP4 *dacB* gene. Within the six isolates with wildtype-resistance levels, five had no mutations that would affect any antimicrobial resistance (AMR) genes of interest when compared to PAO1.

**Conclusion:**

This preliminary study highlights that CZA-resistance in *P. aeruginosa* is multifactorial and could be caused by the interplay between different resistance mechanisms including ESBL carriage, increased efflux, loss of permeability and derepression of its intrinsic *ampC*.

## 1 Introduction

*Pseudomonas aeruginosa* is a leading cause of hospital acquired infections, belonging to the ESKAPE pathogens list, and since 2017 has been indicated as “critical” on the WHO’s priority pathogens list for the research and development of new antibiotics. (World Health Organization, 2017; Horcajada et al., 2019). Ceftazidime-avibactam is one of the last resort antimicrobial agents for the treatment of carbapenem-resistant, Gram-negative bacteria (Simon et al., 2022). Reports on carbapenem-resistant *P. aeruginosa* (CRPA) have increased significantly in recent years and caused much concern, particularly since CRPA infections have been shown to be a significant risk factor for patient mortality (Buehrle et al., 2017). Avibactam (AVI), is a non-β-lactam-based diazabicyclooctane molecule with activity against class A, C and some class D β-lactamases. It is marketed for use in combination with the broad-spectrum cephalosporin ceftazidime, for the treatment of complicated and/or MDR *P. aeruginosa* infections including CRPA (Pfizer, 2019). AVI functions by forming a covalent and reversible bond to the nucleophilic serine of the serine β-lactamases following ring opening, inhibiting the activity of those β-lactamases. Previous studies on the molecular basis of resistance to CZA in *P. aeruginosa*, both on clinical isolates and *in vitro* obtained mutants, have shown that CZA resistance has been associated with the production of metallo-β-lactamases (MBLs)-against which AVI has no activity- altered permeability, increased efflux, and the production of other β-lactamases, including *bla*_PER_ and some class D enzymes (Ortiz de la Rosa et al., 2019; Wang et al., 2020).The overexpression of efflux pump MexAB, in combination with AmpC derepression, has been shown to result in reduced susceptibility to CZA, suggesting that MexAB may play a role in the efflux of AVI (Chalhoub et al., 2018).

This exploratory study sought to examine the mechanisms of ceftazidime-avibactam (CZA) and imipenem (IPM) resistance in a collection of clinical *P. aeruginosa* isolates obtained from Swiss hospitals using a combination of phenotypic testing and whole genome sequencing (WGS).

## 2 Methods

Over a twelve-month period, laboratories from three Swiss Hospitals (Cantonal Hospital of Aarau, University Hospital of Basel, and Cantonal Hospital of St. Gallen) were asked to randomly provide *P. aeruginosa* isolates, which were tested either resistant to CZA or harbored elevated MICs (≥ 4-8 mg/L). In addition, five wild-type *P. aeruginosa* were included. Antibiotic susceptibility testing was performed by either disk diffusion or by broth micro-dilution, according to CLSI methodology (Clinical and Laboratory Standards Institute, 2018), and interpreted according to EUCAST guidelines (EUCAST. Breakpoint Tables for Interpretation of MICs and Zone Diameters, Version 12.0). AmpC and efflux activity were determined by disk testing in the presence/absence of either 2000 mg/L cloxacillin (inhibitor of AmpC activity) or 25 mg/L phenylalanine-arginine β-naphthylamide (PaβN-efflux pump inhibitor) in Muller Hinton agar. Zone diameter differences ≥ 5 mm were considered significant and indicative of AmpC or efflux activity. All susceptibility testing and disk testing experiments were performed in duplicate. Whole genome sequencing (WGS) on 18 clinical isolates was performed as previously described (Findlay et al., 2020). Briefly, WGS was performed by MicrobesNG (https://microbesng.uk/) on a HiSeq 2500 instrument (Illumina, San Diego, CA, USA) using 2x250 bp paired end reads. Reads were trimmed using Trimmomatic (Bolger et al., 2014), assembled into contigs using SPAdes 3.13.0 (http://cab.spbu.ru/software/spades/) and contigs were annotated using Prokka (Seemann, 2014). Resistance genes and sequence types were assigned using the ResFinder and MLST 2.0 on the Centre for Genomic Epidemiology (http://www.genomicepidemiology.org/) platform (Curran et al., 2004; Zankari et al., 2012). Chromosomally encoded genes of interest related to antimicrobial resistance (e.g., efflux genes, porins, as listed in **Supplementary table 1**), as identified in previous studies, were manually extracted from the SPAdes assemblies and Prokka annotation files of sequenced isolates and compared to reference strain *P. aeruginosa* PAO1 (NC_002516.2) (**Supplementary Table 1**).

Sequence data from this study was submitted to the National Center for Biotechnology Information’s Sequence Read Archive (BioProject no. PRJNA914238).

## 3 Results

Eighteen isolates with varying CZA susceptibilities were examined in this study. Eight isolates were CZA resistant with MICs ranging from 16-64 mg/L (**Table 1**). The remaining ten isolates had either MICs that were considered wild-type (1-2 mg/L; n=6) or exhibited elevated but still susceptible MICs (4-8 mg/L; n=4) (EUCAST. Breakpoint Tables for Interpretation of MICs and Zone Diameters, Version 12.0).

**Table 1:** Characteristics of the isolates in this study as identified by susceptibility testing and whole genome sequencing.

			MICs (mg/L)			Disk Testing (mm)					Resistance Mechanisms/Mutations			
Isolate	ST	β-lactamase genes	CAZ	CZA	IPM	CZA	CZA clox	Diameter change (mm)	CZA PaβN	Diameter change (mm)	ESBL Presence	OprD	AmpC-related genes	Efflux genes
PW1	137	OXA-488, PDC-16	4/S	2/S	0.25/S	24	25	1	24	0	N	N	N	N
PW2	395	OXA-905, PDC-8	4/S	2/S	0.25/S	24	26	2	25	1	N	N	N	N
PW3	591	OXA-50, PDC-31	4/S	2/S	0.5/S	23	24	1	24	1	N	N	N	N
PW4	643	OXA-847, PDC-1	4/S	2/S	0.25/S	24	25	1	26	2	N	N	N	N
PW5	179	OXA-396, PDC-8	2/S	1/S	0.25/S	25	25	0	26	1	N	N	N	N
PA338	560	OXA-488, PDC-30	64/R	2/S	1/S	22	27	**7**	27	4	N	N	TR DacB	N
PA915	299	OXA-904, PDC-3	4/S	4/S	0.25/S	23	27	4	20	3	N	N	N	TR NalC
PA862	231	OXA-914, PDC-421	64/R	4/S	16/R	19	25	**6**	20	1	N	TR	Δ*ampDE*, DacB (G115D)	TR MexZ
PA802	2613	OXA-488, PDC-35, OXA-2, PER-1	256/R	8/S	8/R	14	15	1	17	**5**	*bla*_PER-1_	TR	N	TR NalC
PA281	1197	OXA-488, PDC-12	256/R	8/S	1/S	14	20	**6**	15	1	N	N	AmpR (D135N)	N
PA2-R	170	OXA-847, PDC-3	256/R	16/R	8/R	12	22	**10**	15	3	N	N	Δ*ampDE*	N
PA3-R	170	OXA-847, PDC-3	256/R	16/R	32/R	11	19	**8**	13	2	N	TR	Δ*ampDE*	N
PA4-R	155	OXA-396, PDC-5	128/R	16/R	16/R	10	14	4	22	**12**	N	N	N	TR MexR
PA5-R	313	OXA-913, PDC-37	128/R	16/R	16/R	10	18	**8**	19	**9**	N	N	TR AmpD	TR NalD, TR MexZ
PA445	395	OXA-851, PDC-8	128/R	16/R	16/R	15	21	**6**	19	4	N	TR	DacB (G437D)	N
PAVII	181	OXA-486, PDC-3	128/R	16/R	16/R	17	17	0	20	3	N	TR	N	N
PA1-R	2102	OXA-50, PDC-5	128/R	32/R	16/R	11	22	**11**	18	**7**	N	TR	DacB (W388L)	N
PAVI	111	OXA-395, PDC-3, CARB-2	128/R	64/R	16/R	9	13	4	6	0	N	TR	N	N

ST, sequence type; MIC, minimum inhibitory concentration; CAZ, ceftazidime; CZA, ceftazidime-avibactam; IPM, imipenem; CZA clox, ceftazidime-avibactam in presence of 2000 mg/L cloxacillin; CZA PaβN, ceftazidime-avibactam in presence of 25 mg/L PaβN; Zones of inhibition (ZOI) that increased by ≥ 5 mm and were considered significant, are highlighted in **bold**, R, resistant; S, susceptible (according to the EUCAST criteria version 12.0); N, none; TR, truncated; Δ, deleted, parenthesis; amino acid changes.

Sixteen different STs were identified amongst the 18 isolates in this study, all represented by single isolates with the exception of ST170 and ST395, for which there were two representatives each.

Ten allelic variants of the intrinsic, chromosomally encoded *P. aeruginosa* narrow spectrum β-lactamases *bla*_PDC_ (AmpC enzyme), were identified amongst the 18 isolates.

Of β-lactamases, a *bla*_OXA-50-like_ element was found in all isolates comprising of 9 allelic variants respectively. In addition, PA802 (exhibiting a CZA MIC of 8 mg/L) harbored both the narrow spectrum β-lactamase *bla*_OXA-2_ and ESBL *bla*_PER-1_, and PAVI (exhibiting a CZA MIC of 64 mg/L) the narrow spectrum β-lactamase *bla*_CARB-2._ One novel *bla*_PDC_ and four novel *bla*_OXA-50-like_ variants were identified and submitted to the GenBank nucleotide database under accession numbers MT195536 (for *bla*_PDC-421_), and MT210603, MT210600, MT210601, MT210602 (for *bla*_OXA-904_, _-905_, _-913_, and _-914_ respectively). No known carbapenemases were detected in any of the isolates.

Resistance genes of interest (according to **Supplementary Table 1**) were analyzed and mutations or deletions associated with porin deficiency (OprD loss), *ampC* derepression, and increased efflux by the MexAB or MexXY pumps, were found. Notably five isolates exhibiting CZA resistant or elevated MICs, were found to harbor the following mutations, leading to truncated proteins, and linked to efflux upregulation (see **Table 1**): truncations in NalC (PA915 and PA802), NalD (PA5-R) and MexR (PA4-R) which would lead to *mexAB* overexpression. Addition of the efflux pump inhibitor PAβN exhibited a significant effect on isolates PA802, PA5-R and PA4-R. Two isolates (PA862 and PA5-R), harbored a deleterious mutation in *mexZ*, which would lead to *mexXY* overexpression. No significant effect was observed with PAβN in PA862, whereas a zone diameter difference of 9 mm was seen in PA5-R (which in addition harbored a NalD truncation), suggesting that efflux pump MexXY does not play a significant role in CZA resistance.

Concomitant IPM resistance was detected in all isolates that were CZA resistant, including in two isolates with elevated but still susceptible MICs for CZA (**Table 1**). Within the 10 IPM-resistant isolates, seven were found to have mutations in *oprD* resulting in truncations, and 2/3 remaining IPM-resistant isolates with an intact *oprD* harbored deletions or truncations of *ampD* and/or *ampE.*

All eight isolates with mutations related to AmpC hyperproduction, including deletions of *ampD* (PA5-R), *ampDE* (PA2-R, PA3-R, PA862), *ampR* (PA281) mutations and *dacB* mutations or truncations (PA1-R, PA338, PA445, PA862), exhibited significantly increased CZA zones of inhibition in the presence of cloxacillin.

Within the five wild-type isolates (PW 1-5, that were susceptible to ceftazidime, CZA and IPM), no mutations were found that would affect any AMR genes of interest for this study. Overall, within the CZA resistant isolates or those with elevated MICs to CZA, mutations associated with AmpC derepression, OprD loss, *mexAB* overexpression, *mexXY* overexpression and the aforementioned ESBL (*bla*_PER-1_) carriage were observed in various combinations.

## 4 Discussion

We observed a high degree of genomic diversity and ten different allelic variants of the PDC enzyme in the 18 clinical *P. aeruginosa* from three Swiss hospitals. Notably, none of the PDC enzyme variants harbored any of the amino acid changes that have previously been associated with reduced susceptibility to CZA (Slater et al., 2020). Amongst the alleles, one novel *bla*_PDC_ and four novel *bla*_OXA-50-like_ variants were identified.

The increase in CZA zone of inhibition in the presence of cloxacillin in isolates with *dacB* mutations or truncations might be explained by the role of *dacB-* which encodes for penicillin-binding protein PBP4- in triggering derepression of *ampC* relying on a functional AmpR (Moya et al., 2009; Sanz-García et al., 2018). In a previous *in-vitro* study, a G115S mutation could be selected within *dacB* following challenge by ceftazidime, and was linked to *ampC* derepression (Sanz-García et al, 2018). We detected G115D in a ceftazidime resistant isolate that had elevated, but still susceptible MIC to CZA. Further mutations within *dacB* were detected in two isolates (G437D and W388L), both of which showed high-level resistance to ceftazidime and were resistant to CZA. However, the role of *dacB* in CZA-resistance needs further investigation.

The role of *ampC* derepression in CZA-resistance has been evaluated in 26 AmpC mutant *P. aeruginosa* strains *in vitro* by Mushtaq et al. (Mushtaq et al., 2010); Avibactam reversed AmpC-mediated – high-level- ceftazidime resistance in *P. aeruginosa*, reducing MICs for fully derepressed mutants, except in one isolate, that in addition had high-level efflux. On the other hand, Slater et al. could show that AmpC mutants, bearing mutations E247K G183D, T96I, and ΔG229–E247 enhanced the catalytic efficiency of AmpC toward ceftolozane and ceftazidime while simultaneously reducing susceptibility to inhibition by avibactam (Slater et al., 2020). More biochemical studies are needed to elucidate the role of AmpC and CZA-resistance in *P. aeruginosa*.

We did not detect carbapenemases in any of the isolates. In a two-years surveillance of more than 2000 *P. aeruginosa* isolates from the United States, carbapenemases were detected only in 5.9% and 1.0% of 781 IPM- and 119 CZA-resistant isolates respectively (Karlowsky et al., 2022b). The rate of susceptibility for CZA amongst multidrug-resistant (MDR) isolates was 63.3% in *P. aeruginosa.* However, a raise of MIC for CZA amongst all isolates, exceeding the susceptibility-breakpoint, was observed from 2018 to 2020, whereas only a minority of the isolates carried an MBL (Karlowsky et al., 2022b), suggesting adaptive resistance mechanisms as causative for the CZA-resistance. In a 5-year survey, from 2014 to 2019, of more than 7000 P*. aeruginosa* isolates, the annual percent susceptibility rates from North America were higher for CZA (96-99.6%) than for isolates from Europe (90.3-93.1%), 25% of the distinct carbapenem-resistant isolates were also CZA-resistant (Karlowsky et al., 2022a), however, no data was acquired on prevalence of carbapenemases in these specific isolates. Data from more than 4000 *P. aeruginosa* isolates collected from six Latin American countries between 2015 and 2020, revealed a CZA susceptibility rate of approximately 86% amongst all isolates (Wise et al., 2023). Although CZA was the most active antimicrobial agent tested against the subset of multidrug-resistant (MDR) *P. aeruginosa*, their susceptibility was substantially lower with 53.9% in isolates from 2015-2017, dropping to 45.3% in those from 2018-2020, suggesting increased presence of MBLs, against which CZA is inactive (Wise et al., 2023).

One or our isolates exhibiting an CZA MIC at the breakpoint according to EUCAST criteria (EUCAST. Breakpoint Tables for Interpretation of MICs and Zone Diameters, Version 12.0., n.d.), harbored both the narrow spectrum β-lactamase *bla*_OXA-2_ and ESBL *bla*_PER-1_. A recent study has shown that *bla*_PER-1_ alone is capable of conferring CZA-resistance when expressed in a high copy number recombinant plasmid in PAO1 (Ortiz de la Rosa et al., 2019), suggesting that this gene is at least in part responsible for raised CZA MIC observed in our isolate.

We found mutations or deletions associated with porin deficiency (OprD loss), *ampC* derepression, and increased efflux by the MexAB or MexXY pumps. A significant association between CZA-resistance and MexZ disruption was observed in *P. aeruginosa* in a recent study (Castanheira et al., 2019), the authors however concluded, that the role of *mexZ* disruption in CZA-resistance is yet unclear. Despite deleterious mutations in *mexZ*- a transcriptional repressor of the *mexXY* operon (Yamamoto et al., 2009)- no significant effect was observed with addition of the efflux pump inhibitor PAβN –the latter suggesting that the MexXY efflux pump does not play a major role in CZA-resistance. However, efflux pump inhibition exhibited a significant effect in isolates that harbored loss of function mutations in genes that act as transcriptional repressors for the *mexAB-oprM* multidrug efflux operon, namely *mexR* (Cabot et al., 2016) and *nalD* (Braz et al., 2016), indicating a major role of the MexAB-OprM efflux pump in CZA-resistance. The *mexAB-oprM* operon is regulated directly by the transcriptional repressors MexR and NalD and indirectly by NalC (Aguilar-Rodea et al., 2022). This might explain the fact, that efflux pump inhibition revealed significant differences in zone-diameters in the isolates with loss of function of *mexR* and *nalD*, but less so in the isolates with NalC truncation only. These observations are in line with the recent study on 46 CZA-resistant *P. aeruginosa* (Castanheira et al., 2019), in which increased expression of *mexE* and *mexX*, in contrast to MexAB-OprM overexpression, were not significantly associated with CZA-resistance. In addition, MexR disruption and NalD were more frequently observed with CZA-resistant isolates when compared to susceptible counterparts (Castanheira et al., 2019).

### 4.1 Limitations

Our exploratory study has several limitations; the sample size is small and clinical information, such as type of infection and mainly data on antimicrobial pre-treatment were unavailable. Thus we were not analyze these risk factors associated with CZA resistance. Furthermore, complementation assays are needed to prove the role of mutations on resistance. Nevertheless, our data adds to a growing body of evidence on the multifactorial CZA-resistance in *P. aeruginosa*.

## 5 Conclusion

This preliminary study highlights that CZA-resistance in *P. aeruginosa* is multifactorial and can be caused by the interplay between different resistance mechanisms including ESBL carriage, increased efflux, loss of permeability, and derepression of the intrinsic *ampC* whilst IPM-resistance appears to be mainly due to OprD loss as has been extensively reported previously (Trias and Nikaido, 1990; Shu et al., 2017). It is clear that CZA-R in *P. aeruginosa* is multi-faceted and complex, and that we do not yet know all of the mechanisms involved in conferring this phenotype. It is essential that further studies be undertaken to understand how resistance develops to CZA and to identify antibiotics that may promote or induce resistance to CZA, so that steps can be taken to limit the development, thereby extending the life of this increasingly important antibiotic/inhibitor combination.

## Data Availability

The data presented in the study are deposited in the NCBI GenBank repository, accession numbers MT195536 (forblaPDC-421), and MT210603, MT210600, MT210601, MT210602 (for blaOXA-904, -905, -913, and -914 respectively), and WGS data is deposited in the Sequence Read Archive(BioProject no. PRJNA914238).
