# Correction: Multifactorial resistance mechanisms associated with resistance to ceftazidime-avibactam in clinical *Pseudomonas aeruginosa* isolates from Switzerland

**DOI:** 10.3389/fcimb.2025.1636052

**Published:** 2025-07-10

**Authors:** Baharak Babouee Flury, Anja Bösch, Valentin Gisler, Adrian Egli, Salome N. Seiffert, Oliver Nolte, Jacqueline Findlay

**Affiliations:** ^1^ Medical Research Center, Kantonspital St. Gallen, St. Gallen, Switzerland; ^2^ Division of Infectious Diseases and Hospital Epidemiology, Kantonsspital St. Gallen, St. Gallen, Switzerland; ^3^ Department of Infectious Diseases, Inselspital, Bern University Hospital, University of Bern, Bern, Switzerland; ^4^ Clinic of Infectious Diseases and Hospital Hygiene, Kantonsspital Aarau, Aarau, Switzerland; ^5^ Department of Microbiology, Institute for Laboratory Medicine, Kantonsspital Aarau, Aarau, Switzerland; ^6^ Institute of Medical Microbiology, University of Zurich, Zurich, Switzerland; ^7^ Division of Human Microbiology, Centre for Laboratory Medicine, St. Gallen, Switzerland; ^8^ Medical and Molecular Microbiology, Faculty of Science and Medicine, University of Fribourg, Fribourg, Switzerland

**Keywords:** *Pseudomonas aeruginosa*, ceftazidime-avibactam (CZA), molecular resistance mechanisms, imipenem, whole genome sequencing (WGS)

In the published article, there was an error regarding the affiliation(s) for Baharak Babouee Flury. As well as having affiliation(s) 1,2, she should also have “Affiliation 3: Department of Infectious Diseases, Inselspital, Bern University Hospital, University of Bern, Bern, Switzerland.”

The authors apologize for this error and state that this does not change the scientific conclusions of the article in any way. The original article has been updated.

