# Supplementary material for: Multifactorial resistance mechanisms associated with resistance to ceftazidime-avibactam in clinical Pseudomonas aeruginosa isolates from Switzerland
Source: Front Cell Infect Microbiol. 2023 Apr 25;13:1098944. doi: 10.3389/fcimb.2023.1098944 (PMC10166991; doi:10.3389/fcimb.2023.1098944)
Supplement: Supplementary file 1 [file Table1.docx]

**Supplementary table 1**. **Genes analysed by whole genome sequencing**

| Gene of Interest | Role/Associated Phenotype |
| --- | --- |
| *ampD* | AmpC (*bla*_PDC_) regulation/beta-lactam resistance |
| *ampE* |  |
| *ampG* |  |
| *ampR* |  |
| *oprD* | Outer membrane porin/beta-lactam resistance |
| *nfxB* | Efflux genes/extrusion of antibiotics of multiple classes |
| *nalC* |  |
| *nalD* |  |
| *mexA* |  |
| *mexB* |  |
| *mexC* |  |
| *mexR* |  |
| *mexD* |  |
| *mexE* |  |
| *mexF* |  |
| *mexS* |  |
| *mexT* |  |
| *mexX* |  |
| *mexY* |  |
| *mexZ* |  |
| PBP3 | Penicillin-binding proteins/beta-lactam resistance |
| *dacB* |  |
| *mpl* | UDP-N-acetylmuramate; cell wall component recycling |
